# A case series of 223 patients with depersonalization-derealization syndrome

**DOI:** 10.1186/s12888-016-0908-4

**Published:** 2016-06-27

**Authors:** Matthias Michal, Julia Adler, Jörg Wiltink, Iris Reiner, Regine Tschan, Klaus Wölfling, Sabine Weimert, Inka Tuin, Claudia Subic-Wrana, Manfred E. Beutel, Rüdiger Zwerenz

**Affiliations:** Department of Psychosomatic Medicine and Psychotherapy, University Medical Center Mainz, Mainz, Germany

**Keywords:** Depersonalization, Derealization, Health care utilization, Depression, Childhood trauma, Parental history of mental disorders

## Abstract

**Background:**

Depersonalization-derealization syndrome (DDS) is an underdiagnosed and underresearched clinical phenomenon. In Germany, its administrative prevalence is far below the threshold for orphan diseases, although according to epidemiological surveys the diagnosis should be comparable frequent as anorexia nervosa for instance. Against this background, we carried out a large comprehensive survey of a DDS series in a tertiary mental health center with a specialized depersonalization-derealization clinic. To reveal differential characteristics, we compared the DDS patients, who consulted the specialized depersonalization-derealization clinic, with a group of patients with depressive disorders without comorbid DDS from the regular outpatient clinic of the mental health center.

**Methods:**

The sample comprised 223 patients with a diagnosis of depersonalization-derealization-syndrome and 1129 patients with a depressive disorder but without a comorbid diagnosis of DDS. DDS patients were described and compared with depressive outpatients in terms of sociodemographic characteristics, treatment history, treatment wishes, clinical symptomatology, prevailing psychosocial stressors, family history of common mental disorders and history of childhood trauma.

**Results:**

Despite the high comorbidity of DDS patients with depressive disorders and comparable burden with symptoms of depression and anxiety, the clinical picture and course of both patient groups differed strongly. DDS patients were younger, had a significant preponderance of male sex, longer disease duration and an earlier age of onset, a higher education but were more often unemployed. They tended to show more severe functional impairment. They had higher rates of previous or current mental health care utilization. Nearly all DDS patients endorsed the wish for a symptom specific counseling and 70.7 % were interested in the internet-based treatment of their problems. DDS patients had lower levels of self-rated traumatic childhood experiences and current psychosocial stressors. However, they reported a family history of anxiety disorders more often.

**Conclusion:**

In consideration of the selection bias of this study, this case series supports the view that the course of the DDS tends to be long-lasting. DDS patients are severely impaired, utilizing mental health care to a high degree, which nevertheless might not meet their treatment needs, as patients strongly opt for obtaining disorder specific counseling. In view of the size of the problem, more research on the disorder, its course and its optimal treatment is urgently required.

**Electronic supplementary material:**

The online version of this article (doi:10.1186/s12888-016-0908-4) contains supplementary material, which is available to authorized users.

## Background

Depersonalization-derealization syndrome as named in the ICD-10 [1] (or depersonalization-derealization disorder as termed in the DSM-5 [2] is an underresearched clinical phenomenon [3, 4]. Depersonalization-derealization syndrome (DDS) is defined by feeling detached from the own feelings and/or experiences (depersonalization, DP) and/or experiencing objects, people, and/or surroundings as unreal, distant, artificial, and lifeless (derealization, DR) while reality testing remains intact (ICD-10 [1]). Further, symptoms of depersonalization and derealization are not better explained by another mental disorder or medical condition and the symptoms cause significant impairment (DSM-5 [2]). The typical DDS patient, reports that the disorder started before age 25, and that the DP/DR symptoms are present all day long since several years [5–7]. Epidemiological surveys suggest that the current prevalence rate of the depersonalization-derealization syndrome is approximately 1 % in the general population [5–7]. However, the disorder is severely underdiagnosed. For example, in the year 2006 the administrative 1-year-prevalence of the ICD-10 diagnosis “depersonalization-derealization syndrome” was as low as 0.007 % according to the registry of a statutory health insurance fund in Germany [4]. Experts assume this huge diagnostic gap is due to the following reasons: Many clinicians are unfamiliar with the clinical picture and the diagnostic criteria of the disorder. They universally consider symptoms of DP/DR as secondary to a depressive or anxiety disorder, even if these symptoms are all day long present for months and years, or they even misinterpret these symptoms as psychotic although patients are free from any psychotic sings (such as hallucinations, delusions, severe thought disorders, catatonia etc.) [3–5, 8–10]. Moreover, diagnostic awareness is hampered by the patients themselves because many of them are “reluctant to divulge their symptoms out of fears of being thought mad” [8]. Therefore, it usually takes many years from the initial contact with a mental health service until the right diagnosis is made [3, 4, 11].

The current nosological knowledge about the DDS, as it is reported in the recent version of the DSM-5, is largely based on historic descriptions of the disorder, small case-control studies and two descriptive case series with a total sum of 321 patients from specialized clinics or research units in London (UK) and New York (USA). Concerning the etiology of DDS, it has been found that harm-avoidant temperament was associated with DDS in a cross-sectional study [12]. Another cross-sectional study comparing healthy controls with 49 DDS patients demonstrated that emotional abuse was associated with severity of DP/DR but not severe forms of childhood maltreatment [13]. A prospective cohort study found that the only risk factor for severe adult depersonalization at the age of 36 was teacher-estimated childhood anxiety 20 years before. Exposition to environmental risk factors such as socio-economic status, parental death or divorce, and self-reported accidents did not predict later DDS [14]. From an evolutionary perspective, symptoms of DP/DR are considered as a hard-wired response to severe stress, which is perpetuated according to various disease models of DDS by personality factors such as low capacities of self-regulation (e.g., low self-esteem, low affect tolerance, low cohesiveness of the self) [3, 8, 15]. Previous case series from specialized treatment units in London (UK) and New York (USA) reported a sex ratio of 1:1 or even a slight male preponderance [6], with an early age of onset usually before age 25, and a high comorbidity with anxiety and depressive disorders [6, 16]. Both case series demonstrated that the condition had a high chronicity and tended to be resistant to pharmacological and psychotherapeutic treatments [6, 16]. To date, there is no approved medication for the treatment of DDS and there is no randomized controlled trial on the psychotherapeutic treatment of DDS [3].

As the current disease knowledge of DDS has only a small empirical basis, at least as compared to mental disorders with similar prevalence rates and mental health impact, the principle aim of our study was to support and extend the knowledge about the clinical features of the DDS. For that purpose, we examined a large consecutive outpatient sample of DDS patients from the depersonalization-derealization clinic of our department, which has been established in 2005. Patients usually become aware of the clinic by online research about their main complaints (e.g. “feeling unreal”), they are usually self-referred and they typically seek a second opinion regarding their diagnoses and treatment options.

With our study we aimed to address two main questions. Firstly, we sought to describe the typical clinical features and demographic characteristics of patients with DDS as depicted in our clinical standard assessment. Although our case series study is primarily meant as a descriptive study, we included a comparison group form our outpatient clinic in order to bring out the putative differential characteristics of the DDS patients more clearly. For the latter purpose we used a large comparison group of patients suffering from depression without comorbid DDS. We compared both groups in terms of sociodemographic characteristics, treatment history, treatment wishes, clinical symptomatology, level of disability, prevailing psychosocial stressors, family history of common mental disorders, and severity of childhood trauma. We choose a sample of depressed patients for comparison for several reasons: First, this diagnostic entity represents the largest diagnostic group in our department. Second, depression is the most prevalent comorbid condition of DDS patients [6]. Third, depression is a well described and popular disorder thus making it easier for clinicians to acknowledge the similar and differential features of the two groups.

We expected that our case series will constitute an important confirmation and extension of the two previous case series and that it will stimulate further studies on the course, mechanisms and treatment of the disorder.

## Methods

We consecutively included outpatients between January 2010 and December 2013, who consulted the Department of Psychosomatic Medicine and Psychotherapy of the University Medical Center Mainz (Germany). In Germany, Departments of Psychosomatic Medicine and Psychotherapy, usually established at most of all University Medical Centers, are mainly treating patients with depressive disorders, anxiety disorders, somatoform disorders and eating disorders. All patients received a routine psychometric assessment and a clinical interview.

DDS patients who consulted the depersonalization-derealization clinic usually become aware of the clinic by internet research, that is to say, almost all were self-referred. The website of the clinic gives a vivid description of the symptoms and the clinical picture of the disorder. Further, all patients had a short telephone interview with M.M. prior to their consultation, to ensure that they suffer from severe depersonalization/derealization (e.g. as opposed to DP/DR attacks in the context of panic disorder) and to inform them about the focus of the consultation and the therapeutic options of the clinic. Patients from all over Germany were consulting the specialized clinic.

Patients from the comparison group were either self-referred or referred by local physicians and psychotherapist to receive a psychotherapeutic evaluation and treatment recommendations (usually regarding outpatient psychotherapy, inpatient or day clinic psychotherapy). The catchment area of the department is the Rhine-Main-area.

Patients who were treated in the context of the consultation and liaison service (e.g. cancer patients in cancer care units), or who were below age 18 or who had no standardized assessment, or who had no depressive disorder or DDS were excluded.

### Patients

The sample comprised 223 patients with a definite diagnosis of depersonalization-derealization-syndrome (ICD-10: F48.1 [1]) and 1129 patients with a depressive disorder (dysthymia F34.1, or unipolar depression F32.x, or F33.x [1]) but without a comorbid diagnosis of DDS. The latter group will be indicated below as the “Only-Depressed-Group” (ODG). A total of 197 of the 223 patients diagnosed with DDS consulted the depersonalization-derealization clinic of the Department of Psychosomatic Medicine and Psychotherapy of the University Medical Center Mainz, the remaining 26 patients were diagnosed and treated in the general outpatient unit.

### Clinical interview

All patients received a full clinical interview of at least 50 min duration by a psychological or medical psychotherapist. Clinical diagnoses of mental disorders were based on the diagnostic criteria for research of the ICD-10 [1]. The focus of the clinical interview was on the primary presenting problems of the patients and symptom diagnoses. The diagnosis of depersonalization-derealization syndrome was only given, if symptoms of DP/DR were persistent and lasted continuously for at least 1 month and if these symptoms were not better explained by another mental disorder (e.g., unipolar depression, dissociative disorder, anxiety disorder, PTSD) or a medical condition (e.g., seizure disorder). Although the diagnostic criteria of the DDS do not demand specifications about the duration of the symptoms, most clinicians agree that the diagnosis should be only given if the symptoms persist for at least 1 month [3] (see Additional file 1 for comprehensive information about the diagnostic procedure).

Due to the peculiarities of the clinical interview, personality disorders were underreported in our medical records. This was mainly due to the time restriction of the clinical interview. As each patient received a written report about the diagnostic findings, each diagnosis in the record had to be explained to the patient in advance. The diagnosis of personality disorders was rarely made, as most clinicians believed that informing adequately about the diagnosis of a personality disorder requires more time. Because of this bias of underdiagnosing personality disorders in our records, we did not consider personality disorders in this paper.

Further, clinicians rated the social, occupational, and psychological functioning level of psychological functioning by means of the Global Assessment of Functioning (GAF) scale [17, 18]. Lower scores indicate lower levels of functioning. Scores in the range of 51–60 indicate moderate impairment due to symptoms (e.g., flat affect and circumlocutory speech, occasional panic attacks) or moderate difficulty in social, occupational, or school functioning (e.g., few friends, conflicts with peers or co-workers). In Germany, patients with indication for inpatient psychotherapy usually have a current functional level below GAF 50 [19].

### Measures

Severity of DP/DR was assessed with the CDS-2, the two-item version of the Cambridge Depersonalization Scale (CDS [20, 21]). The CDS-2 comprises the following two items of the CDS [22]: “My surroundings feel detached or unreal, as if there was a veil between me and the outside world” and “Out of the blue, I feel strange, as if I were not real or as if I were cut off from the world”. The response format of the CDS-2 was adopted from the Patient Health Questionnaire (“Over the last 2 weeks, how often have you been bothered by any of the following problems?/Not at all = 0/Several days = 1/More than half the days = 2/Nearly every day = 3”). The CDS-2 showed high reliability (Cronbach’s Alpha = 0.92) and was able to differentiate patients with clinically significant DP well from other groups (cut-off of CDS-2 ≥ 3, sensitivity = 78.9 %, specificity = 85.7 %). The CDS-2 sum score (range 0–6) correlated strongly (*r* = 0.77 [22]) with depersonalization severity according to a structured clinical interview of depersonalization severity [23]. Immediately after the CDS-2 items, the patient questionnaire presented the following two questions with a yes/no response: Have you ever consulted a doctor or psychotherapist because of the above symptoms? Do you wish counseling about the above symptoms of depersonalization and derealization?

Severity of depression was measured with the depression module PHQ-9 of the Patient Health Questionnaire [24]. PHQ-9 scores ≥ 10 identified depressive disorders with a sensitivity of 81 % and a specificity of 82 %. Severity of anxiety was measured with the Generalized Anxiety Disorder-7 (GAD-7). The GAD-7 has seven items depicting various signs of generalized and other anxiety disorders (e.g. PTSD, panic disorder). GAD-7 scores range from 0 to 21, with scores of ≥5, ≥10, and ≥15 representing mild, moderate, and severe anxiety symptom levels [25, 26]. The Mini-Social Phobia Inventory (Mini-Spin; [27]) was used for the measurement of social anxiety. The Mini-Spin has three items, which are rated on a 5-point-Likert scale from 0 = “not at all” to 4 = “extremely”. A cut-off score of 6 (range 0–12) separates individuals with social anxiety disorder from controls with good sensitivity (89 %) and specificity (90 %). Somatic symptoms severity was assessed with the 15 items of the PHQ-15. Scores range between 0–30. Scores above 15 identify individuals with high levels of somatic symptom severity respectively somatization severity [28]. The overall mental distress level was measured by the Global Severity Index (GSI) of the German version of the short Symptom Check List (SCL-9) [29]. The range of the GSI is 0 to 4 with higher values reflecting more dysfunction. The ten most common psychosocial stressors (e.g., financial status, family relationships, work, health) were assessed by the corresponding PHQ module on a three-point scale (not bothered = 0, bothered a little =1, bothered a lot =2) [30, 31]. We also calculated the sum score of psychosocial stressors (possible range from 0 to 20). Further, we dichotomized the items (“not bothered” or “little bothered” = 0 versus bothered “a lot” = 1) for the use in a regression analysis. The Childhood Trauma Questionnaire (CTQ) is a 28-item self-report inventory for the assessment of the extent of traumatic childhood experiences. The CTQ has a global score and scores for the subscales emotional abuse, physical abuse, sexual abuse, emotional neglect, physical neglect and minimization [32]. For determining clinically significant levels of traumatization critically cut-points for the subscales have been determined [33, 34]: emotional neglect (≥15), sexual abuse (≥8), physical abuse (≥8), physical neglect (≥10), emotional abuse (≥10). Further, patients gave written information about their socioeconomic details, their treatment history and family history.

### Statistical analysis

Data were presented as mean ± standard deviation, or age and sex adjusted mean, standard error and 95 % confidence interval, or numbers (n) and percentage. Continuous distributed scores were compared by students *T*-test. Categorical variables were compared by Chi-square tests. Associations of continuous data were tested by Pearson correlations. Correlations coefficients of the two groups were compared by the Fisher r-to-z transformation, which controls the correlation coefficients for the effect of different sample sizes. In order to control group differences for the effects of age and sex, we applied logistic regression analyses for binary variables and analysis of covariance (ANCOVA) for continuous variables. In order to evaluate the distinctiveness of the symptom dimensions depression, anxiety, social anxiety and DP/DR we performed a principal component analysis with varimax rotation on the pooled items of the CDS-2, PHQ-9, GAD-7 and Mini-Spin. Tests were considered to be significant at a *p* < 0.05, and all significance tests were two-tailed. Due to the large sample size, the interpretation of the results should focus on effect-sizes rather than *p*-values. SPSS 22.0 was used for the main statistical analysis and VassarStats for the Fisher r-to-z transformation (http://vassarstats.net).

## Results

### Sociodemographic characteristics

Table 1 shows the sociodemographic characteristics of the sample. The group of DDS patients was of younger age and more often male than the “Only-Depressed-Group” (ODG). There was a significant preponderance of men in the DDS group with a female-to-male ratio of 98 to 125 (≈2 : 3). The DDS patients were living less often in a current partnership, were more often still living with their parents, more often holder of the German citizenship, had a higher educational level, but were more often unemployed.

**Table 1:** Sociodemographic characteristics

	DDS *n* = 223	Only Depressed Group *n* = 1129	Test
Age in years	30.7 ± 10.0	40.2 ± 13.0	T = 12.3, *p* < 0.0001
Female sex	43.9 % (98)	61.6 % (696)	*χ* ^2^ = 956.9, *p* < 0.0001
Current partnership (yes)	44.4 % (96)	59.1 % (635)	*χ* ^2^ = 15.8, *p* < 0.0001
Living in the household of the parents (yes)	25.9 % (57)	9.6 % (106)	*χ* ^2^ = 45.2, *p* < 0.0001
Educational level: University-entrance diploma	61.2 % (134)	40.7 % (449)	*χ* ^2^ = 31.2, *p* < 0.0001
Occupational situation			
Employed (part- or fulltime)	33.2 % (74)	46.4 % (524)	*χ* ^2^ = 21.7, *p* < 0.0001
Unemployed	22.1 % (47)	15.1 % (165)	*χ* ^2^ = 6.3, *p* = 0.012
Retirement	4.9 % (11)	9.6 % (108)	*χ* ^2^ = 5.5, *p* = 0.065
German citizenship	95.9 % (211)	91.3 % (1013)	*χ* ^2^ = 5.3, *p* = 0.022

Data are presented as mean ± standard deviation or percentage and numbers in brackets; DDS, patients with depersonalization-derealization syndrome

### Comorbid conditions, symptom burden and clinical course

DDS patients had a very high comorbidity with depressive disorders (84.8 %). As compared with ODG, DDS patients had a higher comorbidity with anxiety disorders, whereas somatoform disorders and PTSD were more prevalent in the ODG. The DDS group had more clinical Axis-I disorders than the controls (2.8 ± 1.0 versus 2.3 ± 1.1, T = 6.920, *p* < 0.0001). Only 21 from 223 DDS-patients (9.4 %) had no comorbid Axis-I disorder. DDS patients had an earlier age of onset and longer disease duration as the ODG (Table 2). DDS had its onset in 63.7 % ≤ age 25, in 17.9 % between age 26 and ≤ 40 and in 4.9 % > 40. There was no valid information about the age of onset for 20 DDS patients.

**Table 2:** The main comorbid conditions and the duration of the main diagnosis

	DDS *n* = 223	Only Depressed Group *n* = 1129	Test
Depressive disorder (F32.-, F33.-, F34.-)	84.8 % (189)	n.a.	
Anxiety disorder (F40.-, F41.-)	42.6 % (95)	30.5 % (344)	*χ* ^2^ = 12.5, *p* < 0.0001
Somatoforme disorder (F45.-)	2.7 % (6)	21.5 % (243)	*χ* ^2^ = 44.0, *p* < 0.0001
Obsessive compulsive disorder (F42.-)	3.1 % (7)	2.9 % (33)	*χ* ^2^ = 0.03, *p* = 0.862
Post traumatic stress disorder (PTSD, F43.1)	0.9 % (2)	6.4 % (72)	*χ* ^2^ = 10.8, *p* = 0.001
Dissociative disorder (F44.-)	1.8 % (4)	3.1 % (35)	*χ* ^2^ = 1.1, *p* = 0.287
Eating disorder (F50.-)	0.4 %(1)	6.8 % (77)	
Disorders due to psychoactive substance use (F1x.-)	4.9 % (11)	7.4 % (83)	*χ* ^2^ = 1.7, *p* = 0.194
Age of onset of main diagnosis	22.9 ± 9.7	35.1 ± 13.4	T = 14.72, *p* < 0.0001
Years since onset of main diagnosis	7.6 ± 7.0	4.6 ± 6.7	T = 5.61, *p* < 0.0001

Comorbid conditions refer to the most prevalent cumulative diagnoses in the sample (excluding personality disorders); n.a.; not applicable; DDS, patients with depersonalization-derealization syndrome

Table 3 shows that after adjustment for age and sex, DDS patients were comparably bothered like the ODG by symptoms of depression (PHQ-9) and anxiety (GAD-7), and they had a similar global severity index (GSI). They had a lower burden with somatic symptoms (PHQ-15) and a slightly lower severity of social anxiety (Mini-Spin). However, severity of depersonalization (CDS-2) strongly separated both patient groups.

**Table 3:** Symptom burden

	DDS (*n* = 223)			Only Depressed Group (*n* = 1129)			ANCOVA Test DDS vs Only Depressed Group		
	Adjusted mean	SE	95 % CI	Adjusted mean	SE	95 % CI	Estimated difference (SE)	95 % CI	*p*-value
Severity of depersonalization (CDS-2)	4.9	0.14	4.7, 5.3	1.9	0.06	1.8, 2.0	3.1 (0.15)	2.8, 3.4	<0.0001
Severity of depression (PHQ-9)	15.3	0.42	14.5, 16.1	16.1	0.18	15.7, 16.4	-0.8 (0.46)	-1.7, 0.1	0.088
Severity of anxiety (GAD-7)	12.3	0.37	11.5, 12.9	12.3	0.16	11.9, 12.6	-0.1 (0.41)	-0.9, 0.8	0.900
Severity of social anxiety (Mini-Spin)	4.9	0.26	4.4, 5.4	5.4	0.11	5.2, 5.6	-0.6 (0.28)	-1.1, -0.0	0.049
Severity of somatoform symptoms (PHQ-15)	11.2	0.39	10.4, 11.9	13.5	0.18	13.1, 13.8	-2.3 (0.44)	-3.2, -1.4	<0.0001
General severity index (GSI) of the SCL-9	1.9	0.06	1.8, 2.0	1.9	0.03	1.9, 2.0	-0.1 (0.07)	-0.2, 0.1	0.190

ANCOVA (df =1), covariates age and sex; data are presented as age and sex adjusted means, standard error (SE) and 95 % confidence interval (95 % CI); DDS, patients with depersonalization-derealization syndrome

In order to evaluate the distinctiveness of the scales we performed a principal component analysis with varimax rotation on the pooled items of the CDS-2, PHQ-9, GAD-7 and Mini-Spin. The Factors were retained in the model based on inspection of the screeplot and eigenvalues > 1. Five factors were identified explaining 61 % of the variance. The items of the CDS-2 were clearly separated from the other scales (data not presented, see Additional file 2). Regarding the association of DP/DR with other symptom dimensions we found that the correlation coefficients of the severity of depersonalization (CDS-2) with anxiety (GAD-7, Mini-Spin), depression, general distress (GSI) and somatization were significantly weaker in the DDS group (Table 4).

**Table 4:** Comparing the strength of correlation of depersonalization severity with anxiety, depression, somatization and general distress

	Pearson correlation of the CDS-2 score		Comparison of the correlation coefficients by Fisher r-to-z transformation
	DDS (*n* = 223)	Only Depressed Group (*n* = 1129)	
Severity of anxiety (GAD-7)	*r* = 0.24***	*r* = 0.41***	z = -2.57, *p* = 0.0102
Severity of social anxiety (Mini-spin)	*r* = 0.11, n.s.	*r* = 0.35**	z = - -3.46, *p* = 0.0005
Severity of depression (PHQ-9)	*r* = 0.25***	*r* = 0.46***	z = -3.25, *p* = 0.0012
Severity of somatization (PHQ-15)	*r* = 0.01, n.s.	*r* = 0.26***	z = -3.44, *p* = 0.0006
Severity of general distress (GSI)	*r* = 0.27***	*r* = 0.47***	z = -3.14, *p* = 0.0017

DDS, patients with depersonalization-derealization syndrome; Fisher r-to-z transformation: Negative z indicates that the correlation coefficient in the DDS group is significantly weaker than that in the ODG; ***, *p* < 0.0001; n.s., not significant; **, *p* < 0.001

### Functional impairment

Both patient groups were markedly impaired by their symptoms (Table 5). After adjustment for age and sex, DDS patients endorsed that their symptoms disrupted their work and social life more strongly than ODG, while the impairment of home life was comparable. These differences were in the range of small to medium effect sizes (Cohen’s d 0.24 to 0.28). Clinicians rated the current and 1-year global level of functioning (GAF) of DDS patients significantly lower than those of the ODG. The difference of GAF was in the range of large effect sizes (Cohen’s d 0.54 to 0.67). Overall, the mean GAF of both groups was in the range of serious to moderate impairment of psychological, social and occupational functioning (GAF 50-60). In the DDS group, 35.2 % had a GAF below 50 which, in Germany, is considered as a criterion for inpatient psychotherapy.

**Table 5:** Functional impairment as self-rated with the Sheehan Disability Scale (SDS) and as clinician rated with the Global Assessment of Functioning scale (GAF)

	DDS (*n* = 217)			Only Depressed Group (*n* = 1027)			ANCOVA Test DDS vs Only Depressed Group		
	Adjusted mean	SE	95 % CI	Adjusted mean	SE	95 % CI	Estimated difference (SE)	95 % CI	*p*-value
SDS: Impairment of work/school	7.9	0.2	7.5, 8.3	7.3	0.09	7.1, 7.4	0.6 (0.2)	0.1, 1.1	0.011
SDS: Impairment of social life	8.1	0.19	7.7, 8.4	7.4	0.08	7.2, 7.6	0.7 (0.2)	0.2, 1.1	0.002
SDS: Impairment of home life	7.2	0.19	6.8, 7.6	6.9	0.09	6.8, 7.1	0.3 (0.2)	-0.2, 0.7	0.256
SDS mean	7.7	0.17	7.4, 8.0	7.2	0.07	7.1, 7.4	0.5 (0.2)	0.1, 0.9	0.006
GAF during the last year	54.5	0.91	52.7, 56.3	59.6	0.40	58.8, 60.3	-5.0 (1.0)	-7.0, -3.1	<0.0001
GAF last seven days	49.7	0.73	48.2, 51.1	51.7	0.32	51.1, 52.4	-2.1 (0.8)	-3.7, -0.5	0.010

ANCOVA (df =1), covariates age and sex; data are presented as age and sex adjusted means, standard error (SE) and 95 % confidence interval (95 % CI); SDS, Sheehan Disability Scale; GAF, Global Assessment of Functioning; GAF during the last year, best level of functioning during the last year; GAF last seven days, indicated the current level of functioning; DDS, patients with depersonalization-derealization syndrome

### Current psychosocial stressors

Overall, DDS patients endorsed being less bothered by psychosocial stressors than the ODG (Table 6). In the sex and age adjusted logistic regression model the following stressors were inversely associated with DDS: weight or appearance worries, difficulties with partners, stress at work or school, financial worries, having no one to turn to, as well as recent or past bad events. The same picture emerged regarding the total burden with psychosocial stressors (i.e. the sum score of the scale): 7.7 ± 3.6 in the DDS group versus 9.7 ± 4.0 in the ODG (T = 7.34, *p* < 0.0001). In the DDS group, there was no correlation between the severity of psychosocial stressors with severity of depersonalization (Pearson correlation between the psychosocial stressor sum score and CDS-2: *r* = 0.06, *p* = 0.39). In the ODG, however, CDS-2 correlated significantly with the sum of psychosocial stressors (*r* = 0.31, *p* < 0.0001). The correlations coefficients differed significantly (Fisher r-to-z transformation: z = 3.53, *p* = 0.0004).

**Table 6:** Association of common psychosocial stressors with Depersonalization-Derealization-Syndrome after multivariate adjustment

“Bothered a lot” by any of the following problems in the last 4 weeks:	DDS (*n* = 223)	Only Depressed Group (*n* = 1129)	Age and sex adjusted OR (95 % CI), p
a) Worrying about your health	61.6 % (135)	63.1 % (685)	1.23 (0.89, 1.69), *p* = 0.204
b) Your weight or how you look	27.3 % (60)	39.9 % (433)	0.93 (0.92, 0.95), *p* = 0.003
c) Little or no sexual desire or pleasure during sex	24.1 % (52)	33.6 % (355)	0.83 (0.58, 1.19), *p* = 0.312
d) Difficulties with husband/wife, partner/lover or boyfriend/girlfriend	18.9 % (39)	33.2 % (345)	0.49 (0.34, 0.73), *p* < 0.0001
e) The stress of taking care of children, parents, or other family members	21.8 % (25)	23.9 % (254)	0.63 (0.40, 1.01), *p* = 0.053
f) Stress at work outside of the home or at school	27.8 % (59)	45.0 % (465)	0.50 (0.36, 0.71), *p* < 0.0001
g) Financial problems or worries	24.5 % (54)	37.2 % (401)	0.59 (0.42, 0.83), *p* = 0.003
h) Having no one to turn to when you have a problem	29.2 % (44)	32.3 % (348)	0.58 (0.40, 0.84), *p* = 0.004
i) Something bad that happened recently	12.7 % (27)	26.6 % (280)	0.48 (0.31, 0.75), *p* = 0.001
j) Thinking or dreaming about something terrible that happened to you in the past …	19.3 % (42)	33.5 % (358)	0.55 (0.38, 0.81), *p* < 0.0001

Data are presented as percentage and numbers in brackets; age and sex adjusted logistic regression analysis, odds ratio (OR), 95 % confidence interval (95 % CI), the stressors were included individually with the covariates age, sex and current partnership; the item a) to j) from the PHQ assessing major psychosocial stressors have been dichotomized (“not bothered” or “little bothered” = 0 versus bothered “a lot” = 1); DDS, patients with depersonalization-derealization syndrome

### Family history and childhood adversities

In the age and sex adjusted regression analysis, only a FH of any anxiety disorder was significantly associated with DDS (Table 7). Regarding childhood adversities, DDS patients showed a similar level of traumatic childhood experiences; only, they endorsed slightly lower levels of physical and sexual abuse than ODG in the age and sex adjusted ANCOVA. Overall, the mean level of traumatic childhood experiences was in the range of minimal to low levels of traumatic childhood experiences (Table 8). Based on the critical cut-points of the CTQ [33, 34], DDS patients reported the following rates of clinically significant levels of traumatization: Emotional abuse 44.7 %, emotional neglect 35.8 %, physical abuse 12.3 %, physical neglect 15.1 %, and sexual abuse 6.1 %. Altogether, 57.8 % of the DDS patients reported at least one significant traumatic childhood experience and 42.2 % none. In the DDS group, there was no association between severity of childhood traumatic experiences with severity of depersonalization (Pearson correlation of the CTQ total score with CDS-2: *r* = 0.05, *p* = 0.44). In the ODG, although weakly, CDS-2 correlated with the CTQ total score (*r* = 0.20, *p* < 0.0001). The correlations coefficients of the two groups differed significantly (Fisher r-to-z transformation, z = 2.07, *p* = 0.0385).

**Table 7:** Family history of major mental disorders

Have your mother or your father ever been diagnosed with one of the following mental disorders?	DDS (*n* = 223)	Only Depressed Group (*n* = 1129)	Age and sex adjusted OR (95 % CI), p
FH of depression	30.7 % (67)	23.0 % (1536)	1.16 (0.83, 1.62), *p* = 0.379
FH of anxiety disorder	14.7 % (32)	7.9 % (157)	1.94 (1.21, 3.11), *p* = 0.006
FH of schizophrenia	1.4 % (3)	1.6 % (31)	0.76 (0.21, 2.73), *p* = 0.76
FH of bipolar disorder	1.8 % (4)	2.5 % (49)	0.67 (0.22, 2.02), *p* = 0.67
FH of any of the above disorders	35.8 % (78)	25.9 % (516)	1.29 (0.94, 1.79), *p* = 0.117

FH, family history; age and sex adjusted logistic regression analysis, odds ratio (OR), 95 % confidence interval (95 % CI); DDS, patients with depersonalization-derealization syndrome

**Table 8:** Severity of traumatic childhood experiences

	DDS (*n* = 223)			Only Depressed Group (*n* = 1129)			ANCOVA Test DDS vs Only Depressed Group		
	Adjusted mean	SE	95 % CI	Adjusted mean	SE	95 % CI	Estimated difference (SE)	95 % CI	*p*-value
CTQ total score	48.8	1.28	46.3, 51.3	47.8	0.43	46.9, 48.6	-1.4 (1.5)	-4.3, 1.6	0.362
CTQ: emotional neglect	12.8	0.39	12.0, 13.6	12.0	0.13	11.8, 12.3	-0.1 (0.4)	-0.9, 0.8	0.891
CTQ: emotional abuse	10.0	0.35	9.3, 10.7	9.3	0.12	9.1, 9.5	0.1 (0.4)	-0.8, 0.9	0.841
CTQ: sexual abuse	5.6	0.22	5.1, 6.0	6.1	0.07	5.9, 6.2	-0.6 (0.3)	-1.1, -0.1	0.023
CTQ: physical abuse	6.1	0.24	5.6, 6.5	6.6	0.08	6.5, 6.8	-0.8 (0.3)	-1.4, -0.3	0.005
CTQ: physical neglect	7.6	0.22	7.2, 8.1	7.7	0.07	7.5, 7.8	-0.2 (0.3)	-0.7, 0.3	0.381
CTQ: denial/minimization	0.5	0.02	0.4, 0.5	0.4	0.06	0.2, 0.5	0.0 (0.1)	-0.1, 0.1	0.909

ANCOVA (df =1), covariates age and sex; data are presented as age and sex adjusted means, standard error (SE) and 95 % confidence interval (95 % CI); CTQ, Childhood Trauma Questionnaire; for the interpretation of the above scores the following cut-points for clinically significant traumatization might be helpful: emotional neglect (≥15), emotional abuse (≥10), sexual abuse (≥8), physical abuse (≥8), physical neglect (≥10); DDS, patients with depersonalization-derealization syndrome

### Treatment history and health care wishes

Overall, DDS had a high treatment rate (Table 9). In the age and sex adjusted regression analysis, previous psychiatric inpatient treatment was much more likely in DDS patients than in the comparison group. The vast majority of the DDS patients endorsed firstly that they had previously consulted a doctor or psychotherapist because of DP/DR symptoms (92.7 % (*n* = 202) versus 25.3 % (*n* = 494)), and secondly that they were interested in DP/DR specific counseling (97.3 % (*n* = 213) versus 35.0 % (*n* = 446)). Those individuals of the ODG, who endorsed the wish for a DP/DR specific counseling, had higher CDS-2 scores than those denying this question (3.1 ± 1.9 versus 0.9 ± 1.3, T = 20.2, *p* < 0.0001). Further, DDS patients more often used the internet for searching information about their symptoms and specialists and were much more interested in internet-based treatment approaches.

**Table 9:** Health care utilization and treatment wishes

	DDS (*n* = 223)	Only Depressed Group (*n* = 1129)	age and sex adjusted OR (95 % CI), p
Current psychotherapeutic treatment	40.4 % (90)	31.8 % (359)	1.29 (0.94, 1.77), *p* = 0.114
Psychiatric inpatient treatment during the last 12 months	20.2 % (45)	4.3 % (48)	4.14 (2.60, 6.59), *p* < 0.0001
Psychosomatic-psychotherapeutic inpatient treatment during the last 12 months	9.4 % (21)	8.1 % (91)	1.27 (0.75, 2.15), *p* = 0.374
Psychiatric or psychosomatic inpatient treatment during the last 12 months	25.6 % (57)	10.8 % (122)	2.62 (1.79, 3.83), *p* < 0.0001
Are you using the internet for searching information about your symptoms?	94.2 % (195)	62.0 % (617)	7.9 (4.3, 14.5), *p* < 0.0001
Are you using the internet for searching specialists for your problems?	84.2 % (176)	58.0 % (585)	3.1 (2.0, 4.6), *p* < 0.0001
Are you interested in an internet-based treatment of your problems?	70.7 % (135)	40.7 % (384)	3.2 (2.3, 4.6), *p* < 0.0001

Data are presented as percentage and numbers in brackets; age and sex adjusted logistic regression analysis, odds ratio (OR), 95 % confidence interval (95 % CI); DDS, patients with depersonalization-derealization syndrome

## Discussion

We investigated a consecutive sample of 223 DDS-patients, who consulted a specialized depersonalization-derealization clinic and compared these patients with a large group of patients with depressive disorders. At the time of the consultation, DDS patients were of younger age, had a significant preponderance of male sex, longer disease duration, an earlier age of onset, and a higher education but they were more often unemployed. Their burden with symptoms of depression and anxiety was comparable, however, they tended to show more severe functional impairment, especially at work/school and in social life. Concerning health care utilization, DDS patients had extraordinary high rates of previous inpatient treatments during the last 12 months (25.6 %) and ongoing outpatient psychotherapy (40.4 %). Despite their high health care utilization, nearly all DDS patients endorsed the wish for a symptom specific counseling (92.7 %) and 70.7 % were interested in an internet-based treatment approach of their problems. With regard to risk factors, DDS patients tended to report lower levels of self-rated traumatic childhood experiences and current psychosocial stressors. However, they more often reported a family history of anxiety disorders. These findings both enhance and extend those of two earlier case series from other countries and health care systems reported by Simeon et al. ([16]) and Baker et al. ([6]).

Very similar to the London case series by Baker et al. ([6]), we found a preponderance of men (125 men to 98 women; Baker et al.: 112 men to 92 women [6]) and almost the same mean age of onset of 22.9 ± 9.7 years (22.8 ± 11.9 years [6]). A similar preponderance of male sex has been recently found for clinically significant DP/DR in a representative questionnaire based survey of pupils in the age of 12 to 18 after adjustment for general distress [35]. The determinants of this putative sex difference in the etiology of DDS warrant further research.

Compared with previous case series, we had a higher proportion of DDS patients reporting an age of onset > 25 years (22.8 %). This finding needs replication, because previous reports assumed that an onset after age of 25 is very rare (less than 5 %) [6, 16]. The larger proportion of DDS patients with a late age of onset in our sample may reflect the increasing use of the internet for health research since 2003, as nearly all DDS patients were referred by themselves or “Dr. Google” respectively.

Similar to Simeon et al. ([16]) and Baker et al. ([6]) the main comorbid conditions were depressive and anxiety disorders. In the current sample only 9.4 % of the DDS in the current sample had no comorbid Axis-I disorder which is very close to 11 % in the case series of Simeon et al. [16]. Despite their high comorbidity and equal symptom burden with symptoms of depression and anxiety, the clinical picture and course of both patient groups differed strongly regarding sociodemographic variables, treatment history and treatment wishes, and risk factors. Again, a principal component analysis substantiated clearly the distinctiveness of DP/DR symptoms from anxiety and depression [36], thus contradicting a commonly held view that symptoms of DP/DR are only a negligible variant of depression and anxiety. The low correlation coefficients of depression or anxiety with DP/DR severity in the DDS group are pointing in the same direction. The much stronger correlation coefficients in the group of the only depressed patients might constitute one reason why many clinicians generally tend to lump together DP/DR symptoms with depression and anxiety. Concerning somatic symptoms severity, DDS patients endorsed significantly less somatic symptoms as compared to the controls. This is in accordance with a recent study, which found that DDS patients endorsed less bodily symptoms of anxiety than pure anxiety patients [37]. The lower burden by bodily symptoms may reflect DDS patients’ detachment from their body.

Although 57.8 % of the DDS patients reported at least one clinically significant traumatic childhood experience, the overall rate of childhood adversities was rather low among DDS patients and even lower than in the comparison group. In line with previous studies [6, 13, 14] this finding makes it unlikely that traumatic childhood experiences play a crucial role in the etiology of DDS. This highlights an apparent contradiction: Although symptoms of DP/DR are typically reactions to severe stress and trauma (e.g. in the case of PTSD [38]), DDS is usually not associated with severe forms of childhood traumatization or recent traumatic events. This suggests that for the development of DDS other factors play a superior role as compared to the exposition to severe traumatic events.

There was a high rate of a parental history of anxiety disorders in the DDS group. Akin to the findings of Baker et al. ([6]), DDS patients had a high rate of psychiatric disorders in a first degree relative (Baker et al.: 30 %; 35.8 % in this sample). This may point to an increased genetic vulnerability of the DDS group on the one hand and on the other hand to an increased environmental risk of being exposed to parents with anxiety disorders [39].

DDS patients endorsed that they were significantly less bothered by current psychosocial stressors than only depressed patients. This either indicates that they have less psychosocial stressors or that they tend to be less aware how psychosocial factors affect them. The latter interpretation would correspond to our clinical experience. Similar to patients with somatoform disorders, DDS patients initially are often unable to consider psychological problems and interpersonal conflicts as relevant causes, and they are convinced by a physical causation of their symptoms [3]. Frequently patients assume a brain tumor, an eye disease or drug induced brain damage as the cause of their symptoms and thus initially consult neurologists, ophthalmologists and other somatic specialists before visiting a mental health specialist [5, 40]. The lack of any correlation between the severity of DP/DR symptoms with the level of current or past stressors might be interpreted in the same way. Severe depersonalization may constitute a “ceiling” effect, which prevents the patients from seeing relations between stressors and their maladaptive stress-response in form of DP/DR. This reminds strongly to a recent study of 291 DDS patients, which found that despite comparable high levels of anxiety, depersonalization and anxiety correlated only in patients with less severe symptoms of DP/DR but not in patients with very high levels of DP/DR [41]. That is to say, therapeutic progress would implicate that patients become aware how the DP/DR symptoms wax and wane depending on the level of the mobilized anxieties [3]. However, in order to test this hypothesis, a longitudinal investigation of these relationships would be necessary.

Making the above considerations, the following major limitations have to be kept in mind. First, our approach implicated a strong selection bias: The DDS-patients were mostly referred by themselves after they have searched the internet for their main complaints, while the comparison group represents patients largely from the near catchment area. This limits the generalizability of our results. For example, we cannot rule out that only DDS patients with a chronic course, poor satisfaction with their current treatment and poor treatment response consulted the depersonalization-derealization clinic of our department. A further bias may constitute the high educational level of the DDS patients. This high educational level could explain the high rate of self-referral among DDS patients coming to a specialized DDS clinic. Highly educated persons may have lower barriers to use the internet for information about health issues. However, the findings concerning chronicity and the high rate of previous health care utilization corresponded well with previous reports from the specialized units in London [6] and New York [16]. Secondly, the diagnoses were based on clinical interviews and not on structured clinical interviews as applied in research settings, thus limiting the validity of our diagnoses. However, diagnoses were enhanced by using the diagnostic research criteria of the ICD-10 and by the correlation of the findings with validated rating scales. Thirdly, family history of mental disorders and history of previous treatments was questionnaire based and not corroborated by independent sources.

## Conclusions

Keeping the above limitations in mind, we found that DDS patients are severely impaired, are utilizing mental health care to a high degree, which nevertheless might not meet their treatment needs, as the patients are taking strong efforts for obtaining symptom specific counseling. This all may reflect the fact that many clinicians are not familiar with the diagnostic features of DDS and its treatment [3]. In Germany, a first step towards the improvement of DDS care may constitute the implementation of the guideline recommendations for the diagnosis and treatment of the depersonalization-derealization syndrome, which have been recently published by the Association of the Scientific Medical Societies in Germany [42]. In view of the size of the problem, much more research on the disorder, its course and its optimal treatment is urgently required.

## Abbreviations

ANCOVA, analysis of covariance; CDS-2, 2-item scale of the Cambridge Depersonalization Scale; CTQ, Childhood Trauma Questionnaire; DDS, Depersonalization-derealization syndrome; DP, depersonalization; DR, derealization; DSM-5, 5th edition of the Diagnostic and Statistical Manual of Mental Disorders; FH, family history; GAD-7, Generalized Anxiety Disorder 7-item scale; GAF, Global Assessment of Functioning; GSI, Global Severity Index; Mini-Spin, Mini-Social Phobia Inventory; OGD, Only-Depressed-Group; OR, odds ratio; PHQ-15, Somatic symptom scale from Patient Health Questionnaire; PHQ-9, depression module of the Patient Health Questionnaire; PTSD, Posttraumatic Stress Disorder; SD, standard deviation; SDS, Sheehan Disability Scale; SE, standard error.

## Additional files

Additional file 1: Additional information about the diagnostic procedure. (DOC 37 kb) Additional file 2: Principal component analysis with varimax rotation of the items of the CDS-2, PHQ-9, GAD-7 and Mini-Spin. (DOCX 21 kb)
