# Supplementary material for: A case series of 223 patients with depersonalization-derealization syndrome
Source: BMC Psychiatry. 2016 Jun 27;16:203. doi: 10.1186/s12888-016-0908-4 (PMC4924239; doi:10.1186/s12888-016-0908-4)
Supplement: Additional file 1: — Additional information about the diagnostic procedure. (DOC 37 kb) [file 12888_2016_908_MOESM1_ESM.doc]

**Supplementary Text A**

This supplementary text explains how the diagnosis of depersonalization-derealization disorder was obtained in the current study. Part 1 summarizes the diagnostic procedure. Part 2 gives the excerpt of the clinical description and diagnostic guidelines according the ICD-10 classification of mental and behavioral disorders [1]. Part 3 describes the procedure of the clinical interview, and part 4 demonstrates the differential diagnostic algorithm.

**Part 1**: The diagnosis of DDS was made, if the patient fulfilled the diagnostic criteria of the depersonalization-derealization syndrome according to the ICD-10, and if symptoms of DP/DR were not better explained by another mental disorder, and if symptoms of DP/DR were the main complaint of the patient, and if these symptoms have lasted continuously for one month at the very least. The typical DDS patient experienced persistent symptoms of DP/DR since several years all day long. Some patients report that the intensity of symptoms, although present all day long, waxes and wanes considerably. Other patients complain that the symptoms are very intense without any change over the time.

**Part 2:** Clinical description and diagnostic guidelines of the depersonalization-derealization syndrome according the ICD-10 classification of mental and behavioral disorders [1]:

**“F48.1 Depersonalization-derealization syndrome**

A disorder in which the sufferer complains that his or her mental activity, body, and/or surroundings are changed in their quality, so as to be unreal, remote, or automatized. Individuals may feel that they are no longer doing their own thinking, imaging, or remembering; that their movements and behaviour are somehow not their own; that their body seems lifeless, detached, or otherwise anomalous; and that their surroundings seem to lack colour and life and appear as artificial, or as a stage on which people are acting contrived roles. In some cases, they may feel as if they were viewing themselves from a distance or as if they were dead. The complaint of loss of emotions is the most frequent among these varied phenomena. The number of individuals who experience this disorder in a pure or isolated form is small. More commonly, depersonalization-derealization phenomena occur in the context of depressive illnesses, phobic disorder, and obsessive-compulsive disorder. Elements of the syndrome may also occur in mentally healthy individuals in states of fatigue, sensory deprivation, hallucinogen intoxication, or as a hypnogogic/ hypnopompic phenomenon. The depersonalization-derealization phenomena are similar to the so-called "near-death experiences" associated with moments of extreme danger to life.

**Diagnostic guidelines**

For a definite diagnosis, there must be either or both of (a) and (b), plus (c) and (d):

(a) depersonalization symptoms, i.e. the individual feels that his or her own feelings and/or experiences are detached, distant, not his or her own, lost, etc;

(b) derealization symptoms, i.e. objects, people, and/or surroundings seem unreal, distant, artificial, colourless, lifeless, etc;

(c) an acceptance that this is a subjective and spontaneous change, not imposed by outside forces or other people (i.e. insight);

(d) a clear sensorium and absence of toxic confusional state or epilepsy.

**Differential diagnosis:** The disorder must be differentiated from other disorders in which "change of personality" is experienced or presented, such as schizophrenia (delusions of transformation or passivity and control experiences), dissociative disorders (where awareness of change is lacking), and some instances of early dementia. The preictal aura of temporal lobe epilepsy and some postictal states may include depersonalization and derealization syndromes as secondary phenomena.

If the depersonalization-derealization syndrome occurs as part of a diagnosable depressive, phobic, obsessive-compulsive, or schizophrenic disorder, the latter should be given precedence as the main diagnosis.”

**Part 2: Description of the clinical procedure during the interview**

During the clinical interview patients were encouraged to give a full and vivid description of their symptoms with their own words. Terse and abstract statements such as “I am depersonalized” or “I feel unreal” were not sufficient. Patients were asked to describe the intensity and duration of their symptoms, and finally their onset and their course. Further, patients were asked to describe whether the intensity of the symptoms is changing other the time and whether an increase or decrease of the intensity of the symptoms was associated with specific situations.

**Part 3: Differential diagnostic algorithm**

As the huge majority of patients with depersonalization-derealization disorder have other comorbid conditions, it is important to find out whether the symptoms of depersonalization and derealization are not better explained by another mental disorder. Regarding this differential diagnostic task, we were guided by the following considerations (for details see the corresponding paragraphs of the DSM-5 [2] and the guideline recommendations for the diagnosis and treatment of the depersonalization-derealization syndrome, which have been recently published by the Association of the Scientific Medical Societies in Germany [3])

Anxiety disorders: In panic disorder the occurrence of DP/DR symptoms is restricted to the short duration of panic attacks. In phobic disorders such as agoraphobia, social phobia or specific phobias the occurrence of DP/DR symptoms is restricted to the increase of anxiety in phobic situations. Regarding generalized anxiety disorder (GAD), symptoms of DP/DR are not the key complaint of the patients, and in GAD these symptoms usually do not occur continuously, i.e. “all day long”, over several months and years. If symptoms of DP/DR are explained by GAD, their occurrence is typically restricted to an increase of anxiety due to worries and forebodings, and the duration of DP/DR symptoms is usually not exceeding several hours or days. In DDS, however, the typical patient is experiencing symptoms of DP/DR all day long for months and years.

Major depressive disorder: In the DDS, the onset of persistent DP/DR symptoms clearly precedes the onset of a depressive episode or clearly continues after the remission of the depressive episode. In addition to the above differential diagnostic criteria, patients with DDS do not only experience numbness, i.e. feeling no emotions, which is very common in severe major depression. However, DDS patients complain the full range of DP/DR symptoms (e.g. “my own voice sounds remote and unreal”; “feeling detached or unreal, as if there were a veil between me and the outside”, “I have the feeling of being outside my body”, “parts of my body feel as if they didn’t belong to me”, “what I see looks ‘flat’ or ‘lifeless’, as if I were looking at a picture”, “I have to touch myself to make sure that I have a body or a real existence” etc. [4]). Further, symptoms of DP/DR clearly constitute the main complaint of patients with DDS.

Psychotic disorder: Patients with DDS have intact reality testing, and they do not consider that the symptoms might be imposed by outside forces or other people, and they are free from delusions, severe thought disorders, hallucinations and catatonia.

Severe dissociative disorders: Patients with DDS have no dissociative amnesia or dissociative identity alterations. If dissociative amnesia or identity alterations are present, the diagnosis of DDS is excluded.

Posttraumatic Stress Disorder (PTSD): The dissociative subgroup of PTSD patients experiences long-lasting symptoms of DP/DR in addition to other key symptoms of PTSD. Contrary to DDS, the onset of PTSD is clearly associated with a severe traumatic event.

Personality disorders: Symptoms of DP/DR belong to the clinical features of the schizotypal personality disorder and the borderline personality disorder. In the case of the Borderline personality disorder, dissociative or paranoid symptoms may occur in episodes of severe stress. In DDS, however, patients experience continuously symptoms of DP/DR for months and years without clear associations with “severe stress”. In the schizotypal personality disorder, symptoms of DP/DR occur as examples of “unusual perceptual experiences including somatosensory (bodily) or other illusions”. The schizotypal personality disorder is characterized by a pattern of extreme discomfort interacting socially, and distorted cognitions and perceptions. The diagnosis of a schizotypal personality disorder usually excludes the diagnosis of DDS.

**References**

1. WHO: The ICD-10 classification of mental and behavioural disorders: clinical descriptions and diagnostic guidelines: Geneva: World Health Organization; 1992.
2. American Psychiatric Association: **Diagnostic and Statistical Manual of Mental Disorders: DSM-5**: American Psychiatric Publishing, Inc.; 2013.
3. Deutsche Gesellschaft für Psychosomatische Medizin und ärztliche Psychotherapie (DGPM), Deutsches Kollegium für Psychosomatische Medizin (DKPM): **Leitlinie: Diagnostik und Behandlung des Depersonalisations-Derealisationssyndroms, Version 1.0 September 2014.** <http://www.awmf.org/leitlinien/detail/ll/051-030.html>. Accessed February 2, 2015
4. Sierra M, Berrios GE: **The Cambridge Depersonalization Scale: a new instrument for the measurement of depersonalization**. *Psychiatry Res* 2000, **93**(2):153-164.
