# Supplementary material for: A case series of 223 patients with depersonalization-derealization syndrome
Source: BMC Psychiatry. 2016 Jun 27;16:203. doi: 10.1186/s12888-016-0908-4 (PMC4924239; doi:10.1186/s12888-016-0908-4)
Supplement: Additional file 2: — Principal component analysis with varimax rotation of the items of the CDS-2, PHQ-9, GAD-7 and Mini-Spin. (DOCX 21 kb) [file 12888_2016_908_MOESM2_ESM.docx]

**Table B:**

**Principal component analysis with varimax rotation of the items of the CDS-2, PHQ-9, GAD-7 and Mini-Spin**

|  | F 1 generalized anxiety | F 2 depression | F 3 social anxiety | F4 DP/DR | F 5 stress/ jumpiness |
| --- | --- | --- | --- | --- | --- |
| CDS-2: Surroundings feel detached or unreal |  |  |  | **0.890** |  |
| CDS-2: I feel strange, as if I were not real |  |  |  | **0.903** |  |
| PHQ-9: Little interest or pleasure | 0.300 | **0.630** |  |  |  |
| PHQ-9: Feeling down, depressed, or hopeless | **0.441** | **0.623** |  |  |  |
| PHQ-9: Sleep trouble |  | **0.642** |  |  |  |
| PHQ-9: Feeling tired or having little energy |  | **0.693** |  |  |  |
| PHQ-9: Poor appetite or overeating |  | **0.569** |  |  |  |
| PHQ-9: Feeling bad about yourself | 0.330 | 0.349 | **0.560** |  |  |
| PHQ-9: Trouble concentrating on things |  | **0.542** |  |  | 0.377 |
| PHQ-9: Psychomotor retardation or agitation |  | 0.319 |  |  | **0.683** |
| PHQ-9: Suicidal ideation |  | **0.405** |  | 0.377 |  |
| GAD-7: Feeling nervous, anxious, or on edge | **0.646** |  |  |  | 0.366 |
| GAD-7: Not being able to control worrying | **0.801** |  |  |  |  |
| GAD-7: Worrying too much | **0.809** |  |  |  |  |
| GAD-7: Trouble relaxing | **0.528** |  |  |  | **0.458** |
| GAD-7: Restless that it's hard to sit still |  |  |  |  | **0.785** |
| GAD-7: Easily annoyed or irritable | 0.336 |  |  |  | **0.482** |
| GAD-7: Something awful might happen | 0.**595** |  |  |  |  |
| Mini-Spin: To avoid doing things or speaking to people. |  |  | **0.818** |  |  |
| Mini-Spin: avoid activities in which I am the center of attention |  |  | **0.766** |  |  |
| Mini-Spin: Being embarrassed or looking stupid are among my worst fears |  |  | **0.835** |  |  |
| Eigen value | 6.9 | 1.8 | 1.6 | 1.3 | 1.2 |
| Explained variance | 32.7% | 8.4% | 7.7% | 6.4% | 5.8% |

Factor loadings > 0.40 are printed in bold types; factor loadings < 0.300 are not shown; the items of the scales are highlighted by different colors: blue CDS-2, grey PHQ-9, yellow GAD-7 and orange Mini-Spin.

Symptoms of depersonalization as measured by the CDS-2 are clearly separated from symptoms of depression, generalized anxiety and social anxiety. However, symptoms as measured by GAD-7, PHQ-9 and Mini-Spin are slightly overlapping.
